# Underlying Resistance Mechanisms in the *Cynosurus echinatus* Biotype to Acetyl CoA Carboxylase-Inhibiting Herbicides

**DOI:** 10.3389/fpls.2016.00449

**Published:** 2016-04-11

**Authors:** Pablo Fernández, Ricardo Alcántara-de la Cruz, Hugo Cruz-Hipólito, María D. Osuna, Rafael De Prado

**Affiliations:** ^1^Department of Agricultural Chemistry and Edaphology, University of CordobaCordoba, Spain; ^2^Bayer CropScience, Col. Ampl. GranadaMéxico; ^3^Agrarian Research Center “Finca La Orden" ValdesequeraBadajoz, Spain

**Keywords:** *Cynosurus echinatus*, ^14^C-DM, metabolism, ACCase activity, ACCase point mutations

## Abstract

Hedgehog dogtail (*Cynosurus echinatus*) is an annual grass, native to Europe, but also widely distributed in North and South America, South Africa, and Australia. Two hedgehog dogtail biotypes, one diclofop-methyl (DM)-resistant and one DM-susceptible were studied in detail for experimental dose-response resistance mechanisms. Herbicide rates that inhibited shoot growth by 50% (GR_50_) were determined for DM, being the resistance factor (GR_50_R/GR_50_S) of 43.81. When amitrole (Cyt. P_450_ inhibitor) was applied before treatment with DM, the R biotype growth was significantly inhibited (GR_50_ of 1019.9 g ai ha^-1^) compared with the GR_50_ (1484.6 g ai ha^-1^) found for the R biotype without pretreatment with amitrole. However, GR_50_ values for S biotype do not vary with or without amitrole pretreatment. Dose-response experiments carried out to evaluate cross-resistance, showed resistance to aryloxyphenoxypropionate (APP), cyclohexanedione (CHD) and phenylpyrazoline (PPZ) inhibiting herbicides. Both R and S biotypes had a similar ^14^C-DM uptake and translocation. The herbicide was poorly distributed among leaves, the rest of the shoot and roots with unappreciable acropetal and/or basipetal DM translocation at 96 h after treatment (HAT). The metabolism of ^14^C-DM, D-acid and D-conjugate metabolites were identified by thin-layer chromatography. The results showed that DM resistance in *C. echinatus* is likely due to enhanced herbicide metabolism, involving Cyt. P_450_ as was demonstrated by indirect assays (amitrole pretreatment). The ACCase *in vitro* assays showed that the target site was very sensitive to APP, CHD and PPZ herbicides in the *C. echinatus* S biotype, while the R biotype was insensitive to the previously mentioned herbicides. DNA sequencing studies confirmed that *C. echinatus* cross-resistance to ACCase inhibitors has been conferred by specific ACCase double point mutations Ile-2041-Asn and Cys-2088-Arg.

## Highlight

Hedgehog dogtail (*Cynosurus echinatus*) is an annual grass that is native to Europe, but is also widely distributed in North and South America, as well as South Africa and Australia. Plants are sensitive to herbicides that selectively inhibit the plastid enzyme ACCase, including diclofop-methyl (DM). Following many years of effective control, some reports have surfaced recently where plants survived lethal doses of DM. A hedgehog dogtail population in Chile was confirmed resistant to DM in 1999. In addition to exhibiting high levels of resistance to DM, cross-resistance to other ACCase inhibitors was also documented. The underlying basis for resistance has been ascribed to two separate mechanisms: enhanced metabolism of DM resulting from elevated activity of Cytochrome P_450_ monooxygenase; and a double mutation in a target site region of ACCase, resulting in Ile-2041-Asn and Cys-2088-Arg amino acid substitutions.

## Introduction

Hedgehog dogtail (*Cynosurus echinatus* L.) is an annual grass, native to Europe, widely distributed in North and South America, South Africa, and Australia (Boersma et al., 2006). Selective and effective control of hedgehog dogtail in wheat was only possible with the ACCase diclofop-methyl (DM) introduced at the beginning of the 90s into Chile. The first case of herbicide resistance in hedgehog dogtail in the world was reported in 1999 in Chile to DM and clodinafop (Espinoza and Diaz, 2005; Bakkali et al., 2007; Valverde, 2007; Heap, 2016). The potential cross-resistance of diclofop-resistant hedgehog dogtail populations to these most recently commercialized herbicides had not been documented. Cross resistance refers to the resistance of an individual or a population to multiple herbicides due to a single resistance mechanism such as target site mutation and/or increased metabolism (Heap, 2016). These herbicides do not necessarily belong to the same chemical family such as the APPs (DM, fenoxaprop-butyl and clodinafoppropargyl), CHDs (clethodim, sethoxydim, cycloxydim), and PPZ (pinoxaden) (De Prado and Franco, 2004; Kaundun, 2014; Yu and Powles, 2014; Cruz-Hipolito et al., 2015).

Graminicide herbicides inhibit the chloroplastic acetyl coenzyme A carboxylase (ACCase; EC.6.4.1.2) action in the Poacea family, preventing fatty acid synthesis and reducing the production of the phospholipids used in the membranes (Deley et al., 2005). Chlorosis, necrosis, and finally the death of plant tissue occur after applying these herbicides (Ball et al., 2007). Repeated use of acetyl-CoA carboxylase inhibiting herbicides, APP, CHD, and PPZ, has resulted in the evolution of resistance in 47 grass weed species worldwide (Heap, 2016). There are two mechanisms of resistance to graminicide herbicides: that caused by mutation(s) in the gene encoding the herbicide target protein (Kaundun, 2010; Petit et al., 2010; Cruz-Hipolito et al., 2011, 2012, 2015; Scarabel et al., 2011) and/or to other mechanism(s) causing a reduction in the amount of herbicide molecules reaching their target (such as enhanced metabolism, foliar penetration, translocation, and others; Shimabukuro and Hoffer, 1992; Letouzé and Gasquez, 2003; De Prado et al., 2005; Yuan et al., 2007; Powles and Yu, 2010; Kaundun, 2014). Lately, Han et al. (2016) have demonstrated that, in different *Lolium rigidum* populations collected in Australia, both resistance mechanisms of metabolism and ACCase DM resistance mutation(s) may occur.

The objectives of this research were to determine the resistance patterns of *C. echinatus* biotypes to DM, and to other ACCase inhibitors (APP, CHD, and PPZ), and to elucidate the mechanisms behind their resistance from foliar penetration, metabolic, ACCase assays and molecular basis for resistance to cross-resistance inhibitors in this biotype.

## Materials and Methods

### Chemicals

[2, 4-dichlorophenoxy-U-^14^C]-diclofop-methyl (specific activity, 95.5 kBq μmol^-1^) was provided by Bayer CropScience (Germany).

The following herbicides and reagents were used in this study: technical grade fenoxaprop-acid; cyhalofop-acid; diclofop-acid; sethoxydim; cycloxydim; and pinoxaden. All other reagents was obtained at analytical grades.A commercial herbicide formulation were used for dose-response assays in a greenhouse: Puma Super, 5.5% fenoxaprop-p-ethyl w:v EC; Clincher, 20% cyhalofop-butyl w:v EC; Iloxan, 36% DM w:v EC; Focus Ultra, 10% cycloxydim w:v EC; Poast, 20% setoxydim w:v EC; Axial, 10% pinoxaden w:v EC. The etizol herbicide (24% amitrole w:v SL), was used as a Cyt. P_450_ inhibitor.

### Plant Material

In 2012, hedgehog dogtail (*C. echinatus*) seeds collected were not controlled with DM and cycloxydim at doses normally used in a wheat cropped field located in the region of “La Arauconia” (38–39° latitude Southern) in Chile. Subsequently, farmers switched to pinoxaden alone to gain better control of these grasses. After several years of implementation of these new herbicides, farmers began to notice a lesser efficiency. Seed collection was conducted on 50 plants which had reached physiological maturity and they were subsequently dried at laboratory temperature of 25°C for 2 weeks. Later, they were stored in paper bags in a 4°C chamber. In 2013, approximately 500 seeds of this species were sown directly onto trays 40 cm wide × 60 cm long × 15 cm deep, containing a mixture substrate of sand:peat (2:1, v:v) and placed in a greenhouse at 28/20°C day/night under a 16 h photoperiod with 850 μmol m^-2^ s^-1^ photon flux density, and 80% relative humidity. When *C. echinatus* plants showed 3–4 leaves, pinoxaden was applied at 300 g ai ha^-1^ using a laboratory spray chamber equipped with a flat fan nozzle (TeeJet 8002 EVS). The equipment was calibrated to give 300 L ha^-1^ at a pressure of 200 kPa. Four hours after application, trays containing plants were carried out to the greenhouse and watered daily. Four weeks after the pinoxaden application, visual assessment (0: no injury; 100: dead plants) was performed, those dead plants and those that had over 50% damage were discarded. Survivor plants matured normally and were finally collected, dried and stored in paper bags for all subsequent trials and named as R.

The *C. echinatus* seeds proposed for this study were collected from nearby areas (“Del Bio Bio,” 36–38° latitude Southern) which had never been treated with herbicide. However, for safety’s sake, 500 seeds of this specie were planted as described above for R population. When each plant reached the 3–4 leaf state, it was treated with pinoxaden at 50 g ai ha^-1^ as described above. The visual assessment after 4 weeks of pinoxaden application showed that all plants were dead and were considered as being susceptible (S) to the herbicide.

### Growth Assays and Herbicide Treatments

Seeds of R and S biotypes of *C. echinatus* were germinated on moistened filter paper in Petri dishes. Seedlings were planted in 8 cm × 8 cm × 10 cm pots (three plants per pot) as explained above. At 3–4 leaf stage, R and S biotypes of *C. echinatus* were sprayed with different herbicides and doses. The doses (g ai ha^-1^) of herbicide applied were the following: cyhalofop-butyl (0, 50, 100, 200, 300, 600, 700, 1000, 2000, 3000, and 4000); DM (0, 25, 50, 100, 200, 250, 500, 1000, 2000, and 3000); fenoxaprop-p-ethyl (0, 25, 50, 100, 200, 500, 1000, 2000, 3000, and 4000); cycloxydim (0, 10, 20, 40, 60, and 100); sethoxydim (0, 10, 20, 30, 40, 60, and 80); pinoxaden (0, 4, 8, 16, 32, 64, 128, 256, and 512).

Above-ground fresh weight per pot was determined 21 days after treatment (DAT), and data were expressed as the percentage of the untreated control. Herbicide rates inhibiting plant growth by a 50% decrease in growth with respect to the untreated control (GR_50_) were determined for each biotype, and the R/S ratio (FR) was computed as GR_50_(R)/GR_50_(S) (Seefeldt et al., 1995). Data were pooled and fitted to a non-linear, log-logistic regression model:

$$Y=c+\left\{\left(d-c\right)/\left[1+{\left(x/g\right)}^{b}\right]\right\},$$

where *Y* is the fresh above ground weight expressed as the percentage of the untreated control, *c* and *d* are the coefficients corresponding to the lower and upper asymptotes, *b* is the slope of the line, *g* is the herbicide rate at the point of inflection halfway between the upper and lower asymptotes, and *x* (independent variable) is the herbicide dose. Regression analysis was conducted using the Sigma plot 10.0 statistical software. The experiment was repeated twice in a completely randomized design with five replications per dose.

### Diclofop-Methyl Growth Assays in Combination with Cyt. P_450_ Inhibitor

Amitrole has previously been shown to inhibit DM in *L. rigidum* metabolism (Preston and Powles, 1998). Our preliminary experiments showed that amitrole applied with amounts up to 100 to 200 g ai ha^-1^ had not an adverse toxic effect on the seedling growth of *C. echinatus*. Seedling of R and S biotypes at the 2–3 leaf stage were treated with DM at doses as shown above, with (200 g ai ha^-1^) or without amitrole. Amitrole was applied 8 h prior to DM application. The experiment was repeated twice with three replicates (nine technical replications for each biotype).

### Foliar Uptake and Translocation of ^14^C-DM

^14^C-DM was mixed with commercially formulated DM to prepare an emulsion with a specific activity of 37.9 Bq mg^-1^ and a DM concentration of 6.6 g L^-1^ (corresponding to 1.0 kg ha^-1^ DM at 150 L ha^-1^). This formulation of labeled herbicide was applied to the adaxial surface of the second leaf in each plant in four 0.5 μL droplets by means of a Hamilton PB-600 microapplicator. A total of 833.33 Bq was applied to each plant.

*Cynosurus echinatus* R and S plants were harvested in batches of three after variable lengths of time (12, 24, 48, 72, and 96 h) following the application of the herbicide and split into treated leaves, the remainder of the shoots, and roots. Unabsorbed ^14^C-DM was removed from the leaf surface by washing the treated area with 1.5 mL of acetone. Washes from each batch were pooled and analyzed by liquid scintillation spectrometry (LSS) on a Beckman LS 6000 TA instrument. Plant tissue was dried at 60°C for 48 h and combusted in a sample oxidizer (Packard 307). Evolved ^14^CO_2_ was trapped and counted in 14 mL of Carbosob/Permafluor E^+^ (7/7 V/V; Packard Instruments Co.). Radioactivity was quantified by LSS and expressed as a percentage of recovered radioactivity, using the following expression:

% absorption=[C⁢ in⁢ combusted⁢ tissue14C⁢ in⁢ combusted⁢ tissue+C⁢ in⁢ leaf⁢ washes)1414]×100.

The experiment was repeated three times.

For translocation tests, ^14^C-DM was applied to the second leaf as described above. At intervals of 12, 24, 48, 72, and 96 h after herbicide application, the treated (second) leaf, untreated (first and third) leaves and roots were harvested separately. The treated leaf was rinsed and unabsorbed radiolabel quantified by LSS as described above. The treated leaf and untreated leaves and roots were oven-dried at 60°C for 2 days, combusted in a sample oxidizer as described above, and analyzed for radioactivity by LSS. Percent diclofop translocation was expressed as:

$$\left[\frac{k\,B_{q}\,\;i\,n\,\;s\,h\,o\,o\,t\,\;t\,i\,s\,s\,u\,e\,\;o\,u\,t\,s\,i\,d\,e\,\;\;t\,h\,e\,\;t\,r\,e\,a\,t\,e\,d\,\;l\,e\,a\,f}{\left(k\,B_{q}\,\;i\,n\,\;r\,i\,n\,s\,e\,d\,\;t\,r\,e\,a\,t\,e\,d\,\;l\,e\,a\,f+k\,B_{q}\,\;i\,n\,\;s\,h\,o\,o\,t\,\;t\,i\,s\,s\,u\,e\,\;o\,u\,t\,s\,i\,d\,e\,\;\;t\,h\,e\,\;t\,r\,e\,a\,t\,e\,d\,\;l\,e\,a\,f\right)}\right]\times 100.$$

The experiment consisted of five replicates per harvest time and biotype, arranged in a completely randomized design.

### Phosphor Imaging

A phosphor imager was used to observe ^14^C-translocation (Cyclone, Perkin-Elmer, Packard Bioscience BV). Plants were treated with respective unlabelled and radiolabelled DM as described for foliar uptake and translocation assays. Whole plants were rinsed and oven-dried (60°C, 4 days); pressed plants were placed adjacent to 25 cm × 12.5 cm phosphor storage film during 10 h and scanned for radiolabel dispersion. The experiment was replicated three times per biotype.

### Enzyme Purification and ACCase Assays

The ACCase enzyme was partially isolated according to Cruz-Hipolito et al. (2011, 2012). Leaves (6 g fresh weight) of R and S biotypes of *C. echinatus* were harvested from plants in 3–4 leaf stages and ground in liquid N_2_ in a mortar and then added to 24 ml of extraction buffer (0.1 M N-2-hydroxyethylpiperazine-*N′-2*-ethanesulfonic acid-KOH [pH 7.5], 0.5 M glycerol, 2 mM EDTA, and 0.32 mM PMSF). The homogenate was mixed for 3 min with a magnetic stirrer and filtered sequentially through four layers of cheesecloth and two of Miracloth. The crude extract was centrifuged (24000 *g*, 30 min, 4°C). The supernatant was fractionated with (NH_4_)_2_SO_4_ and was centrifuged (12000 *g*, 10 min, 4°C). Material precipitating between 35 and 45% (NH_4_)_2_SO_4_ saturation was re-suspended in 1 ml of S400 buffer [0.1 M Tricine-KOH (pH 8.3), 0.5 M glycerol, 0.05 M KCl, 2 mM EDTA, and 0.5 mM DTT]. The clarified supernatant was applied to a desalting column previously equilibrated with S400 buffer. The ACCase enzyme was eluted from the column in 2 mL S400 buffer.

The enzyme activity was assayed by measuring the ATP-dependent incorporation of NaH[^14^C]O_3_ into an acid-stable [^14^C]-product. The reaction product has been previously shown to be [^14^C]-malonyl-CoA (Parker et al., 1990). Assays were conducted in 7 mL scintillation vials containing 0.1 M tricine-KOH (pH 8.3), 0.5 M glycerol, 0.05 M KCl, 2 mM EDTA, and 0.5 mM DTT, 1.5 mM ATP, 5 mM MgCl_2_, 15 mM NaH[^14^C]O_3_ (1.22 MBq μmol^-1^), 50 μl enzyme fraction, and 5 mM acetyl-CoA in a final volume of 0.2 mL. Activity was assayed for 5 min at 34°C, and the reaction was stopped by adding 30 μl of 4 N HCl. A piece of filter paper was added to the reaction vial and the sample was dried at 40°C under a stream of air. After the sample was dried, ethanol-water (1:1, v:v, 0.5 ml) was added to the vial, followed by the addition of 5 ml of scintillation cocktail. Radioactivity was determined by LSS. Background radioactivity, measured as acid-stable counts (kBq) in the absence of acetyl-CoA, was subtracted from each treatment. One unit of ACCase activity was defined as 1 μmol malonyl CoA formed min^-1^. ACCase inhibiting herbicide concentrations resulting in a 50% inhibition of enzyme activity (*I*_50_) were determined in crude extracts. Data were pooled and fitted to the log-logistic model described previously. Experiments were repeated twice with five replicates per biotype.

### Metabolic Study with ^14^C-DM

Herbicide metabolism was studied in leaf tissue from *C. echinatus* R and S plants at the 2–3 leaf stage as in the penetration studies. A labeled herbicide was applied to the adaxial surface of the second leaf in 10 0.5 μL droplets by using a microapplicator. A total of 5000 Bq was used on each plant. Plants of the R and S biotypes were sampled 12, 24, 48, 72, and 96 h after treatment (HAT). Treated leaves were washed as described above. An aliquot of leaf wash solution was assayed for radioactivity, and the remaining solution stored at -20°C until analysis. Treated plants were split into shoots and roots were discarded. The shoots from each plant were powdered in liquid nitrogen by using a mortar and pestle. The powder was extracted with 4 mL of 80% methanol at 4°C and the homogenate centrifuged at 20 000 *g* for 20 min. The resulting pellet was washed with 80% methanol until no further ^14^C was extracted. The pellets were oven-dried and combusted as described above. The supernatants were combined, evaporated to dryness at 40°C under a stream of N_2_ at 10 kPa and re-dissolved in 500 μL of 80% methanol. DM and its metabolites in the supernatant were identified by thin-layer chromatography on 20 cm × 20 cm, 250 μm silica gel plates (Merck; silica gel 60), using a 150/7/7 v/v/v toluene/ethanol/acetic acid mixture as mobile phase. Radioactive zones were detected with a radiochromatogram scanner (Berthold LB 2821) and their chemical nature was identified by comparing their *R*_f_ values with those for standards (DM, 0.70; diclofop acid, 0.44; hydroxy-diclofop, 0.34; polar conjugates, 0.00). The experiment was arranged in a completely randomized design with five replications per biotype.

### Metabolic Study with^14^C-DM in Combination with Cyt. P_450_ Inhibitor

At 3–4 leaf stage, R and S biotypes of *C. echinatus* were sprayed with amitrole at 200 g ai ha^-1^ as explained in growth assays. After 8 h of applying amitrole, the DM metabolism was performed following the method previously proposed at 12, 24, 48, 72, and 96 HAT.

### DNA Extraction and ACCase Amplification

Seedlings at the 3–4 leaf stage were treated with pinoxaden at a rate of 300 g ai ha^-1^. Foliar tissue (50 mg) of individual plants from the resistant and susceptible biotypes was taken before treatment for use in the molecular analysis. This rate eliminated all susceptible population plants 21 DAT (Cruz-Hipolito et al., 2011, 2012; Hatami et al., 2016).

DNA from the leaf fragment was extracted using the Speedtools Plant DNA Extraction Kit (Biotools B&M Labs S.A., Spain), following manufacturer’s instructions, and DNA amount was quantified in a NANODROP Thermoscientific spectrophotometer (ThermoFisher, Nano- Drop Products, Wilmington, DE, USA). Each DNA sample was diluted until a final concentration of 10 ng/μl, which was immediately used for the polymerase chain reaction (PCR) or stored at -20°C until use.

Two pairs of primers were designed to amplify regions in the CT domain known to be involved in sensitivity to ACCase herbicides (**Table 1**). Two sets of primers covering all five known mutation sites in region A (1781; primers AC1781F/R) and B (2027, 2041, 2078, and 2096) (primers AC2F/R), were designed based on the chloroplastic ACCase sequences of other grass weeds, *Alopecurus myosuroides* (accession no. AJ310767), *Bromus diandrus* (AJ966446), *Hordeum vulgare* (AJ966456), *L. multiflorum* (AY710293), *L. rigidum* (AY995232), *Zea mays* (U19183). Ten individual plants from each biotype were genotyped.

**Table 1:** Primers used to amplify the ACCase gene.

Primers	Sequence (5–3′)
AC1781F	CTGCAGCTGGATAGTGGTGA
AC1781R	AAGCTTGTTCAGGGCAGAAA
AC2F	AGCTTGGAGGAATCCCTGTT
AC2R	GGGTCAAGCCTACCCATACA

ACCase gene fragments were amplified in a thermal cycler, with a final volume of 20 μl, containing 10 ng of template DNA, 0.75 μM of each primer, 1.6 μM of dNTPs, 2 μl of PCR buffer and five units of *Thermus aquaticus* (Taq) DNA polymerase. The PCR amplifications were performed with the following profile: 1 cycle of 94°C 2 min; 35 cycles of 30 s at 94°C, 30 seg at 58°C (for AC1781F/R primers) or at 60°C (for AC2F/R primers), and 1 min at 72°C; followed by a final extension cycle of 5 min at 72°C.

Amplified DNA fragments were purified using the Speed tools PCR Clean-Up Kit (Biotools, B&M Labs, Madrid, Spain). Sequencing of the purified genomic DNA was done in the Genomic Unit Investigation Central Service of Badajoz University, Spain.

## Results

### Growth Assays and Herbicide Treatments

The calculated GR_50_ values and the R/S ratios of both *C. echinatus* biotypes in response to several ACCase inhibitor herbicides are shown in **Table 2**. The resistance to DM was confirmed in the R biotype (GR_50_ = 1484.6 g ai ha^-1^) compared with the values found by the S one (GR_50_ = 33.9 g ai ha^-1^). The DM reduced the fresh weight of S biotype at low doses and eliminated plants at up to a dose of 500 g ai ha^-1^, whereas in the R biotype the same dose had no effect on the fresh weight and plants just died at up to a dose of 4000 g ai ha^-1^. The APP presented the highest FR values (fenoxaprop-p-ethyl > diclofop-methyl > cyhalofop-butyl > propaquizafop), while the R biotype has a high resistance to PPZ (pinoxaden), the cross-resistance corresponding to CHD (cycloxydim > setoxydim) herbicides being low (**Table 2**).

**Table 2:** Equation parameters of the sigmoidal curve used to calculate the dose of herbicide required to reduce 50% of the fresh weight (GR_50_) of *Cynosurus echinatus* R and S biotypes, and the ratios obtained (FR resistance factor) of resistant biotypes.

Herbicide	Biotype	Maximum	Minimum	Hill slope	*R*^2^	GR_50_ (g ai ha^-1^)	FR
Fenoxaprop-p-ethyl	S	98.97	1.09	0.74	0.94	45.56	58.16
	R	100	4.90	6.69	0.99	2650.28	
Cyhalofop-butyl	S	99.99	1.07	0.83	0.99	200.97	19.93
	R	99.94	8.62	2.11	0.94	2800.79	
Diclofop-methyl	S	99.61	3.41	0.75	0.97	33.89	43.81
	R	91.69	12.04	5.38	0.99	1484.58	
Setoxydim	S	98.27	1.18	1.74	0.97	13.87	2.18
	R	99.99	3.62	3.29	0.99	30.27	
Cycloxydim	S	99.99	5.56	0.85	0.98	12.66	3.19
	R	99.97	7.26	1.97	0.99	40.42	
Pinoxaden	S	99.74	3.87	1.64	0.99	16.25	16.64
	R	99.99	5.36	2.87	0.98	270.47	

*Data were fit by a non-linear regression model. *Y* = Minimum + {(Maximum–Minimum)/[1 + (x/GR_50_)^∗Hillslope^]} where *Y* represents the maximum and minimum fresh weight expressed as a percentage of weight relative to untreated plants correspond to the upper and lower asymptotes, Hill slope, and the independent variable x is the dose of herbicide*.

### Growth Assays in Combination with Cyt. P_450_ Inhibitor

**FIGURE 1:**
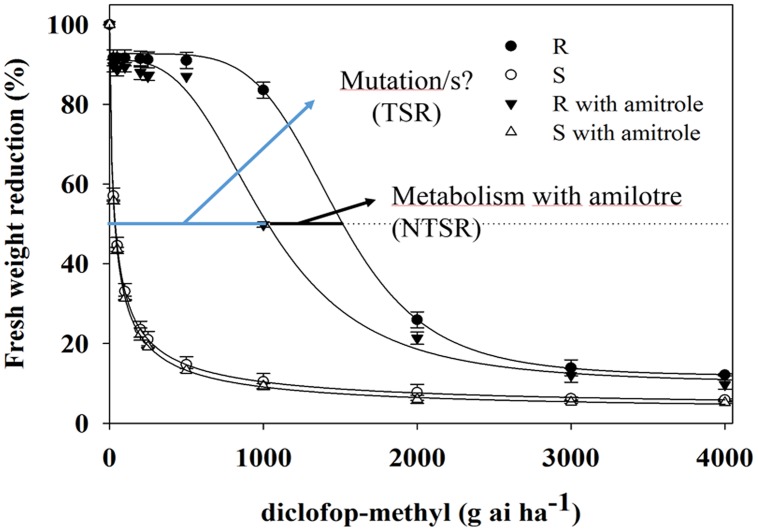
**Diclofop-methyl (DM) dose response of *Cynosurus echinatus* R and S biotypes, comparing the effect amitrole pretreatment (R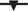; S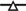 and nor pretreatment (R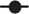; S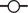)**. Symbols denote mean ± standard error of the mean.

The growth response to DM showed marked differences between *C. echinatus* biotypes (**Figure 1**). The pretreatment with amitrole significantly inhibited the growth of R biotypes (GR_50_ of 1019.9 g ai ha^-1^) compared with the GR_50_ (1484.6 g ai ha^-1^) found for the R biotype without pretreatment with amitrole. However, GR_50_ values for the S biotype did not vary and this was independent of amitrole treatment (**Figure 1**). The RF values were higher in plants (R) without amitrole (RF = 43.8) compared with those found in plants (R) pretreated with amitrole (RF = 31.4), indicating that Cyt. P_450_ was involved in DM-resistance in *C. echinatus*. However, reducing the resistance level by 28.3% cannot be explained only by the metabolism of the herbicide to non-toxic forms and (an)other additional mechanism(s) should be studied.

### ^14^C-DM Foliar Uptake, Translocation, and Plant Visualization

**FIGURE 2:**
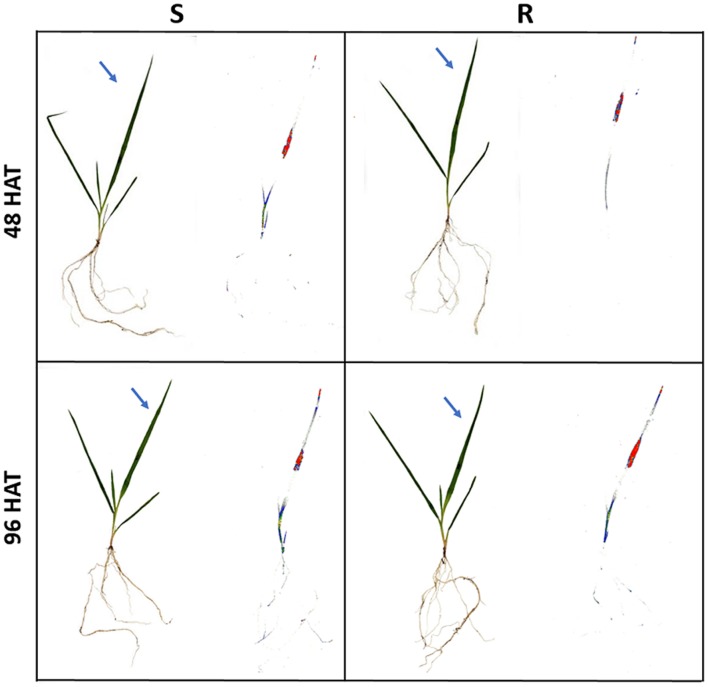
**Phosphor images of *C. echinatus* R and S biotypes treated with ^14^C-DM at 48 and 96 h after treatment (HAT)**.

In general, more than 93.0 ± 2.5% of the total applied radioactivity was recovered in the uptake, translocation, and metabolism study. Foliar uptake of ^14^C-DM increased with time in both *C. echinatus* R and S biotypes (**Table 3**). The ^14^C-DM peak foliar uptake occurred at 72 and 96 HAT for R and S plants. However, the S biotype seemed to reach the maximum peak penetration faster than the R biotype. Taken as a whole, foliar absorption levels did not seem to be involved in resistance between biotypes. Similarly, ^14^C-DM uptake and translocation were confirmed through phosphorimaging in both R and S biotypes (**Figure 2**). The herbicide was poorly distributed among leaves, roots and the rest of the shoot with unappreciable acropetal and/or basipetal DM translocation at 96 HAT. The picture taken through the phosphorimaging could not detect the difference in DM translocation from the treated leaf to the root in either biotype of *C. echinatus* found in the above test (**Table 3** and **Figure 2**). These results suggest that an altered symplast transport is not responsible as a mechanism for DM-resistance in *C. echinatus*.

**Table 3:** ^14^C-DM uptake and translocation (% of absorbed) in *C. echinatus* R and S biotypes at different time applications^a^.

Biotype	HAT	% Uptake	Translocation (% of absorbed)		
			Treated leaf	Shoots	Roots
Susceptible	12	12.2 ± 2.1^g^	84.0 ± 3.6^ab^	16.0 ± 3.4^ab^	0.0
	24	38.4 ± 3.6^e^	80.6 ± 4.0^ab^	18.1 ± 2.2^ab^	1.3 ± 0.5^cd^
	48	63.7 ± 4.7^d^	77.1 ± 3.3^abc^	19.6 ± 3.7^ab^	3.3 ± 1.1^abc^
	72	86.7 ± 3.0^b^	73.6 ± 2.5^bc^	21.7 ± 4.1^ab^	4.6 ± 1.5^ab^
	96	90.6 ± 4.2^a^	68.6 ± 3.1^c^	25.6 ± 3.0^a^	5.6 ± 1.3^a^
					
Resistant	12	8.1 ± 1.6^h^	86.2 ± 3.6^a^	14.2 ± 4.0^b^	0.0
	24	34.0 ± 3.2^f^	84.4 ± 2.9^ab^	15.4 ± 2.6^b^	1.0 ± 0.3^cd^
	48	65.7 ± 2.5^d^	81.5 ± 4.8^ab^	17.3 ± 4.0^ab^	1.2 ± 0.7^cd^
	72	83.3 ± 4.1^c^	79.7 ± 4.0^abc^	19.6 ± 3.1^ab^	2.1 ± 0.8^cd^
	96	88.2 ± 5.6^ab^	76.1 ± 3.6^abc^	22.4 ± 2.9^ab^	1.7 ± 0.5^cd^

*^a^The experiment was carry out in triplicate. Means (*n* = 8) ± SE. Means in a column followed by the same letter were not significantly different at the % probability level as per Tukey’s test*.

### ^14^C-DM Metabolism Alone or in Combination with Cyt. P_450_ Inhibitor

**FIGURE 3:**
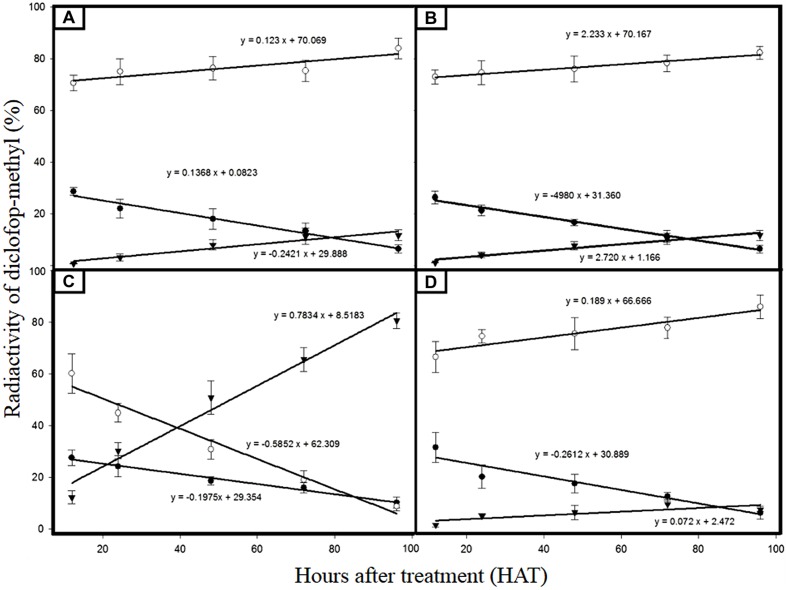
**Radioactivity percentage of DM and its metabolites from *C. echinatus* plants. (A)** Susceptible biotype (without amitrole); **(B)** Susceptible biotype (with amitrole); **(C)** Resistant biotype (without amitrole); and **(D)** Resistant biotype (with amitrole). DM (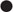); D-acid (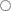); D- conjugate (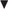).

Metabolites (DM, D-acid and D-conjugate) were found in both *C. echinatus* R and S biotypes (**Figures 3A,C**). However, DM was de-esterified to D-acid significantly faster and greater in the S than in the R biotype, while D-acid remained nearly constant (80%) in S biotype over the time (from 12 to 96 HAT), although in the R biotype it dropped to below 8.8% at 96 HAT (**Figures 3A,C**). This is because in the R biotype, monooxygenases Cyt. P_450_ quickly formed a D-hydroxylated metabolite and then a nontoxic D-conjugate. Although this route was also followed by the S biotype, the formation speed of the D-conjugate was slower (0.24) than of the R biotype (0.78; **Figures 3A,C**).

Foliar pretreatment with amitrole significantly inhibited DM metabolism to the polar D-conjugate in R biotypes (**Figures 3C,D**), but not in S biotypes, where the polar D-conjugate went on being minimum (**Figures 3A,B**).

### ACCase Assays

The ACCase *in vitro* assays showed that the target site was very sensitive to APP, CHD, and PPZ herbicides in the *C. echinatus* S biotype, while de R biotype was insensitive to the former (**Table 4**). Based on the obtained resistance factors (FR), it was clear that there was a cross- resistance to the three ACCase inhibitor herbicide groups studied (**Table 4**). Evidently, the R biotype of *C. echinatus* was highly resistant to pinoxaden (PPZ), moderately resistant to fenoxaprop, diclofop, and cyhalofop (APP) and poorly resistant to setoxydim and cycloxidim (CHD) herbicides. The results suggest a less sensitive form of ACCase in the R biotype than in the susceptible biotype, determining this characteristic cross-resistance to ACCase inhibiting herbicides. The resistance in the *C. echinatus* R has been related to a mutation in the ACCAse gene diminishing the target site sensitivity to the herbicides.

**Table 4:** Equation parameters of the sigmoidal curve used to calculate herbicide concentration required to reduce 50% of the ACCase activity (*I*_50_) of *C. echinatus* R and S biotypes and the ratios obtained (FR resistance factor) of resistant biotypes.

Herbicide	Biotype	Maximum	Minimum	Hill slope	*R*^2^	I_50_ (μM)	RF
Fenoxaprop	S	99.63	5.32	0.86	0.99	1.58	15.3
	R	100.00	6.61	0.90	0.99	24.18	
Cihalofop	S	100.00	3.24	0.89	0.99	10.19	6.9
	R	100.00	1.08	0.51	0.97	70.77	
Diclofop	S	100.00	2.13	0.78	0.99	4.53	14.0
	R	100.00	3.03	0.41	0.98	63.46	
Setoxidim	S	98.34	1.07	0.69	0.97	800.11	4.3
	R	98.39	4.07	0.81	0.98	3500.93	
Cycloxidim	S	99.01	1.70	1.61	0.99	2.5	3.1
	R	97.05	3.17	1.46	0.98	7.89	
Pinoxaden	S	99.99	0.79	0.78	0.99	0.39	23.3
	R	99.98	9.27	1.23	0.98	9.1	

*Data were fit by a non-linear regression model. *Y* = Minimum + {(Maximum-Minimum)/[1 + (x/I_50_)^∗Hillslope^]} where *Y* represents the maximum and minimum ACCase activity expressed as a percentage of ACCase activity relative to untreated plants correspond to the upper and lower asymptotes, Hill slope, and the independent variable x is the dose of herbicide*.

### ACCase Gene Sequencing

**FIGURE 4:**
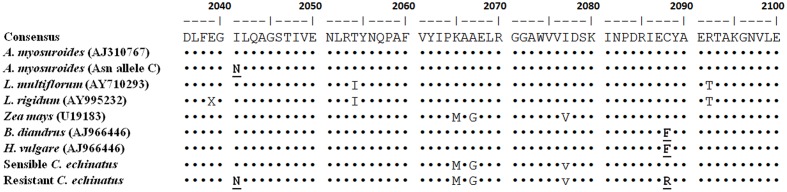
**Alignment of partial amino acid sequences of chloroplastic homomeric ACCase from various grass species**. The substitution in the resistant biotype of *C. echinatus* from Chile is shown in bold.

Two fragments of 229 and 478 bp of the CT domain of the ACCase gene corresponding to the A and B region, respectively, were sequenced. Single nucleotide polymorphisms (SNPs) were found in the coding sequence. *C. echinatus* showed six non-synonymous SNP’s respect to *A. myosuroides* corresponding to the 1789, 2041, 2065, 2067, 2077, and 2088 positions (**Table 5**). The 1789, 2065, 2067, and 2077 positions were considered unrelated to resistance. The sequences obtained of *C. echinatus* were aligned to each other and with the CT domain of plastidic ACCase genes of other grass weeds (**Figure 4**). The nucleotide sequences of the A region between the R and S biotype did not differ. In the B region, the R biotype showed two SNP’s compared to S biotype resulting in a double substitution in the target-site. A codon change from ATT to AAT resulted in an amino acid substitution of Ile-2041-Asn, whereas, a codon change from TGT to CGT resulted in an amino acid substitution of Cys-2088-Arg in the R biotype (**Table 5**). No other mutations were found (data not shown).

**Table 5:** Nucleotide sequence and predicted amino acids of ACCase DNA isolated from susceptible and resistant populations of *C. echinatus* compared to *Alopecurus myosuroides* (GenBank Accession no. AJ310767).

Aminoacid position^∗^	1789	2041	2065	2067	2077	2088
*A. myosuroides* (AJ310767)	AGT/Ser	**ATT/Ile**	AAG/Lys	GCA/Ala	ATT/Ile	**TGC/Cys**
*C. echinatus* S	AGA/Arg	**ATT/Ile**	ATG/Met	GGC/Gly	GTT/Val	**TGT/Cys**
*C. echinatus* R	AGA/Arg	**AAT/Asn**	ATG/Met	GGC/Gly	GTT/Val	**CGT/Arg**

*^∗^Columns in bold indicate the positions considered related to resistance*.

## Discussion

In recent years, cross-resistance to ACCase-inhibiting herbicides in some grass weeds has become a serious threat to wheat production in Chile (Espinoza and Diaz, 2005). The results found by us for this species are in good agreement with other results that report different levels of resistance and cross-resistance patterns of various grasses resistant to the three groups ACCase inhibiting herbicides (Tal et al., 1996; De Prado et al., 2004; Maneechote et al., 2005; Cruz-Hipolito et al., 2011, 2012). A variety of compounds (ABT, amitrole, PBO, malathion) have proven to be effective in inhibiting herbicide metabolism in plants catalyzed by Cyt. P_450_ (Preston, 2004; De Prado et al., 2005; Powles and Yu, 2010; Yu et al., 2012; Kaundun, 2014; Pan et al., 2015). In this current study, pretreatment with amitrole decreased the GR_50_ value of DM by 28% in the R biotype, whereas it does not vary and is independent of amitrole treatment in the S biotype, suggesting that an enhanced metabolism mediated by Cyt. P_450_ monooxygenase may also play a role in DM-resistance in the R biotype. It is widely accepted that DM is rapidly de-esterified by hydrolysis in crops and weeds to produce D-acid, which is the actual active ingredient (De Prado et al., 2005; Yu et al., 2012). Later, D-acid is metabolized to mainly sugar conjugates of hydroxy-diclofop, which is mediated by cytochrome P_450_ monooxygenases (P_450_′S), this compound being more polar than D-acid and not at all phytotoxic (Preston and Powles, 1998; Preston, 2004; De Prado et al., 2005). In agreement with other author who have used Cyt. P_450_ inhibitors (amitrole and ABT), the amount of nontoxic D-conjugates formed 96 HAT in R biotypes was reduced by over 10 times the amount of D-conjugate metabolites observed in R biotypes not preincubated with amitrole (De Prado et al., 2005; Yu et al., 2012; Han et al., 2016). These results suggest that the aryl-*O*-glucoside was the major component of the conjugated fraction because the D-hydroxylated metabolite was strongly inhibited by the amitrole action (Holtum et al., 1991; Menendez and De Prado, 1996; De Prado et al., 2005; Kaundun, 2014).

Previous research, in general, has demonstrated that differences in foliar uptake between R and S biotypes have not been involved in Poaceae weed resistance to DM (Menendez and De Prado, 1996; De Prado and Franco, 2004; De Prado et al., 2005; Cruz-Hipolito et al., 2011, 2012). A similar distribution of ^14^C-DM (or any metabolites formed thereafter) in R and S biotypes was found when addressing the recovered radioactivity. Over time, small differences in the translocation from the treated leaf to the roots were detected in the S biotype with respect to the R one. However, these results did not imply considering uptake and translocation as a determining DM-resistance mechanism mainly due to the large amount of herbicide remaining in the treated leaf (**Table 3**). These results are in agreement with others obtained in our laboratory (De Prado et al., 2005; Cruz-Hipolito et al., 2011, 2012, 2015; Fernandez et al., 2015) and other international groups (Kaundun, 2014; Yu and Powles, 2014) working with grasses and concluded that there is a poor DM distribution throughout the plant.

Resistance due to target gene mutation has been profoundly studied in the world (Heap, 2016). The species resistant to ACCase inhibitor herbicides most widely investigated have been: *Lolium* spp, A*vena* spp, *Alopecurus* spp, and *Phalaris* spp (Yu et al., 2010; Cruz-Hipolito et al., 2011, 2012; Kaundun, 2014; Fernandez et al., 2015). Generally, most of them are resistant to APP and CHD herbicides, and, in very few cases, cross-resistance to APP, CHD, and PPZ (**Table 4**). Previous cases of APP and CHD herbicide resistance in grasses correlating with reduced sensitivity in the target enzyme have been reported. Target site resistance is essentially caused by a single amino acid change in the CT domain, which impacts the effective binding of ACCase-inhibiting. Seven different codons (1781, 1999, 2027, 2041, 2078, 2088, and 2096) responsible for resistance have been described in grassy weeds (Délye et al., 2008; Powles and Yu, 2010; Kaundun, 2014; Vila-Aiub et al., 2015). The Ile1781Leu substitution is the most predominantly known to cause resistance to most APP herbicides. It has been shown that biotypes with this mutation exhibit resistance to fops (APP), dims (CHD) and pinoxaden (PPZ) (Liu et al., 2007; Délye et al., 2008; Beckie and Tardif, 2012). One of the mutations found in this work (Ile-2041-Asn) contributes to the fop-binding site, which explains the strong effect of the Ile to Asn substitution in the loss of sensitivity to these herbicides (Jang et al., 2013). Previous studies have demonstrated that the other aminoacid change found in this work (Cys-2088-Arg) confers resistance to APP, CHD and PPZ herbicides (Yu et al., 2007; Xu et al., 2013). It has been suggested that the Cys-2088-Arg substitution is located in the CT-active site (Xu et al., 2013). Then, two amino acid differences, Ile-2041-Asn and Cys-2088-Arg, are sufficient to explain resistance of *C. echinatus* biotype to most of the herbicides tested in this work for the first time in the world.

This study is the first documented case where two mechanisms (RST and NRST) are involved in cross-resistance to ACCase inhibiting herbicides in hedgehog dogtail collected in Chile. The resistance is associated with an enhanced metabolism mediated by Cyt. P_450_ monooxygenase and two point mutations (Ile-2041-Asn and Cys-2088-Arg) at the CT domain of the ACCase gene. As a consequence this herbicide group is becoming ineffective in *C. echinatus* control and farmers and technicians must be very careful with the use of selective alternative herbicides with different action mechanisms because Cyt. P_450_ monooxygenase is unpredictable and can trigger herbicide resistance with similar mechanisms or even different action mechanisms, including herbicides never used. Farmers should consider the integration of a pasture phase, which allows the use of grazing and the use of non-selective herbicides in order to prevent the weed seed productions. An integrated weed management (IWM) is required to allow the reduction and elimination of herbicide-resistant handling populations.

## Author Contributions

PF, and HC-H performed the DM plant response and ACCase activity assays; RA, PF, and RD performed ^14^C-DM absorption/translocation, and ^14^C-DM metabolism; RA, and MO performed the ACCase gene sequencing.

## Conflict of Interest Statement

The authors declare that the research was conducted in the absence of any commercial or financial relationships that could be construed as a potential conflict of interest.
